# Editorial: Novel Two-Dimensional Energy-Environmental Materials

**DOI:** 10.3390/molecules31030463

**Published:** 2026-01-29

**Authors:** Guangzhao Wang, Xiangkai Kong, Ning Wang

**Affiliations:** 1 Chongqing Key Laboratory of Optical Chip and Intelligent Optoelectronic Systems, Chongqing Key Laboratory of Extraordinary Bond Engineering and Advanced Materials Technology, School of Electronic Information Engineering, Yangtze Normal University, Chongqing 408100, China; 2 School of Materials Science and Physics, China University of Mining and Technology, Xuzhou 221116, China; 3 Key Laboratory of High Performance Scientific Computation, School of Science, Xihua University, Chengdu 610039, China

Two-dimensional (2D) materials and heterostructures, benefiting from their ultrathin thickness [1], high strength [2], excellent conductivity [3], tunable electronic structure [4], and high specific surface area [5,6], demonstrate great potential in the fields of energy and environmental applications [7,8,9,10,11,12,13,14]. They provide new avenues for addressing energy shortages and environmental pollution.

This Special Issue focuses on the design, applications, and key research developments regarding innovative 2D materials and heterostructures in the areas of energy and environmental materials. It comprises a collection of 11 articles covering diverse areas, including energy conversion and storage, environmental protection and pollution control, and also the design of magnetic materials. Overall, these findings aim to open up new avenues for the development of related materials and mark a critical step towards their practical application.

## 1. Energy Conversion and Storage

Electrocatalytic hydrogen production is a promising approach for addressing the energy crisis. However, developing inexpensive and efficient catalysts remains challenging. Using first-principles calculations, Chen et al. [15] studied vacancy-defective Janus WSSe monolayers. They found that vacancy defects lower the hydrogen evolution reaction (HER) Gibbs free energy and enhance activity, as explained by p-band center theory. Additionally, biaxial strain could effectively tune HER performance via adaptive bond relaxation at defect sites. This study provides theoretical guidance for designing efficient HER catalysts based on defective Janus transition metal dichalcogenides (TMDs). Developing suitable hydrogen storage materials is also a major challenge for the hydrogen energy industry. Huang et al. [16] used density functional theory (DFT) calculations to investigate the TiB_7_ cluster, revealing its structural stability under near-ambient conditions. The Ti atom enables the dissociative absorption of H_2_, accommodating up to five H_2_ molecules with a storage capacity of 7.5 wt.%. The average hydrogen adsorption energy of 0.27–0.32 eV suggests reversible hydrogen storage under mild conditions, governed by both polarization and hybridization mechanisms. Achieving stable and highly efficient lead-free 2D perovskite materials is still a major challenge. Long et al. [17] utilized first-principles calculations to predict the 2D perovskite CsTeI5, which exhibits excellent stability and a theoretical power conversion efficiency (PCE) as high as 29.3%, making it a promising candidate for thin-film solar cells. Zhao et al. [18] investigated the mechanical and thermal transport properties of 2D Si-Ge lateral heterostructures. Their mechanical behavior can be effectively tuned by applying strain, introducing vacancy defects, and varying the temperature. For thermal transport, non-equilibrium molecular dynamics simulations reveal that the effective phonon mean free paths are 136.09 nm for zigzag interfaces and 194.34 nm for armchair interfaces. This research provides a theoretical basis for applying Si-Ge lateral heterostructures in thermal management devices.

## 2. Environmental Protection and Pollution Control

Adsorption is a cost-effective water purification method and its performance relies on material design. Dotti et al. [19] employed reduced graphene oxide (rGO) and vine pruning biochar (VBC) to fabricate an rGO/biochar composite membrane using a green process. With an optimized mass ratio of 4:6, the membrane exhibited rapid adsorption of Cu^2+^ and Zn^2+^ within 10 min, with maximum capacities of 4.03 mmol g^−1^ and 21.99 mmol g^−1^, respectively. Its overall performance indicates that effective integration of the characteristics of both components and demonstrates significant application potential. Liao et al. [20] synthesized In_2_S_3_/CuInS_2_ microflower heterojunctions with sulfur vacancies (Vs-In_2_S_3_/CuInS_2_) using an In_2_S_3_ microsphere template followed by a Cu^+^/In^3+^ ion exchange strategy. Under visible light, the optimized heterostructure exhibits clearly enhanced photocatalytic activity, with CO and CH_4_ production rates (80.3 and 11.8 μmol g^−1^ h^−1^) 4 and 6.8 times greater than those of pure In_2_S_3_. The enhanced photocatalytic activity originates from three key factors: the heterojunction structure that promotes charge separation, the uniform microflower morphology that improves light harvesting and electron transport, and the S vacancies that suppress charge recombination. In their review, Li et al. [21] highlight the distinct catalytic roles of precious metals and more cost-effective alternative metals in the glycerol valorization. The focus is on the mechanisms through which bimetallic and metal oxide catalysts enhance performance via synergistic effects, involving key processes such as selective adsorption, oxidation, and C-C bond cleavage. Finally, they outline future directions for developing efficient and highly selective glycerol catalytic systems through innovative catalyst design, in-depth mechanistic studies, and sustainable process scale-up.

## 3. Magnetic Material Design

Two-dimensional spin-gapless magnetic materials open avenues for merging spin polarization with the quantum anomalous Hall effect (QAHE). Hu et al. [22] identified monolayer h-VN as a promising spin-gapless semiconductor (SGS) to overcome the typical limitations of low Curie temperature and constrained topological control, achieving full spin polarization and a high Curie temperature (~543 K). It exhibits a tunable topological gap under spin–orbit coupling (SOC), showing a quantum anomalous Hall state (Chern number C = 1) at SOC strengths up to 250%. This property remains robust under strain and when loaded on an h-BN substrate. This system offers a practical platform for high-temperature QAHE physics. Zhao et al. [23] used first-principles calculations to predict that the *Pca21* C_4_N_3_ monolayer material possesses a ferromagnetic ground state and exhibits SGS characteristics, with a notably smaller bandgap in the spin-down channel than in the spin-up channel. The adsorption of CO molecules (CO@C_4_N_3_) could effectively tune the electronic structure, and stable adsorption selectively narrows the spin-down band gap. Moreover, hole injection increases the net magnetic moment and triggers an SGS-to-metal transition, whereas electron injection reduces the magnetic moment and triggers an SGS-to-half-metal transition. This work provides valuable insights for designing practical metal-free SGS materials for spintronics. Zhao et al. [24] attributes the magnetism of the *Pca21* C_4_N_3_ monolayer to spin splitting in the *2p_z_* orbitals of key C atoms and the *2p* orbitals of adjacent N atoms near the Fermi level. The magnetic moment can be effectively tuned by gas adsorption. Chemisorption of NO reduces the magnetic moment from 4.00 μB to 2.99 μB by forming Nsub-Nad bonds, whereas physisorption of O_2_ increases the magnetic moment to 6.00 μB via ferromagnetic coupling. This reversible, simple, and cost-effective gas adsorption control method establishes an important theoretical basis for developing low-dimensional magnetic materials. Ma et al. [25] prepared large-area, thickness-controllable 2D Cr_7_Te_8_ nanosheets via chemical vapor deposition. Cr_7_Te_8_ exhibits strong perpendicular magnetic anisotropy even at thicknesses of only a few layers, with a thickness-dependent Curie temperature that ranges from 180 to 270 K. The anomalous Hall resistance shows a sign reversal with temperature, originating from changes in Berry curvature. Furthermore, a low-temperature topological Hall effect reveals nontrivial spin chirality. These results demonstrate the potential for controlling topological magnetic states in two-dimensional ferromagnets, and pave the way for designing novel spintronic devices.

This Special Issue aims to offer a comprehensive overview of advanced materials for energy, environmental, and spintronic applications. We hope that this Special Issue will provide valuable insights into advanced materials being developed for energy conversion and storage, environmental protection and pollution control, and spintronics. Finally, we thank all of the authors, reviewers, and editors for their invaluable contributions to this collection.

## Data Availability

The data presented in this study are available upon request from the corresponding authors.
